# Predictive Analytic Techniques to Identify Hidden Relationships between Training Load, Fatigue and Muscle Strains in Young Soccer Players

**DOI:** 10.3390/sports10010003

**Published:** 2021-12-24

**Authors:** Mauro Mandorino, António J. Figueiredo, Gianluca Cima, Antonio Tessitore

**Affiliations:** 1 Department of Movement, Human and Health Sciences, University of Rome ‘Foro Italico’, 00135 Rome, Italy; m.mandorino@studenti.uniroma4.it; 2 Research Unit for Sport and Physical Activity, Faculty of Sport Sciences and Physical Education, University of Coimbra, 3040-248 Coimbra, Portugal; afigueiredo@fcdef.uc.pt; 3 Computer, Control and Management Engineering Department, Sapienza University of Rome, 00185 Rome, Italy; cima@diag.uniroma1.it

**Keywords:** youth soccer, workload, injury, fatigue, predictive analytics

## Abstract

This study aimed to analyze different predictive analytic techniques to forecast the risk of muscle strain injuries (MSI) in youth soccer based on training load data. Twenty-two young soccer players (age: 13.5 ± 0.3 years) were recruited, and an injury surveillance system was applied to record all MSI during the season. Anthropometric data, predicted age at peak height velocity, and skeletal age were collected. The session-RPE method was daily employed to quantify internal training/match load, and monotony, strain, and cumulative load over the weeks were calculated. A countermovement jump (CMJ) test was submitted before and after each training/match to quantify players’ neuromuscular fatigue. All these data were used to predict the risk of MSI through different data mining models: Logistic Regression (LR), Random Forest (RF), Support Vector Machine (SVM). Among them, SVM showed the best predictive ability (area under the curve = 0.84 ± 0.05). Then, Decision tree (DT) algorithm was employed to understand the interactions identified by the SVM model. The rules extracted by DT revealed how the risk of injury could change according to players’ maturity status, neuromuscular fatigue, anthropometric factors, higher workloads, and low recovery status. This approach allowed to identify MSI and the underlying risk factors.

## 1. Introduction

Most of the injuries occurring in youth soccer primarily involve lower extremities, and among them muscle strains are more frequently reported (~35%) [1,2]. Beyond the economic impact on the National Health System [3], muscle strains may lead to several physical consequences, especially in young athletes. Indeed, an injury may determine long-term sequelae, time-loss from sport participation [4], and a higher probability to incur in a re-injury in the future [5], jeopardizing the talent development processes. Muscle strains are a complex multifactorial phenomenon. Among the numerous factors, fatigue, defined as the transient reduction in the ability to produce force or power [6], may play a crucial role in the onset of muscle injuries. Indeed, previous studies on youth football reported a higher incidence of muscle strains during the last 15 min of each half of a competitive match [7,8,9]. Muscle fatigue, impairing neuromuscular control and dynamic stability, may increase susceptibility to injury [10]. Moreover, fatigued muscles absorb less energy before failure compared to non-fatigued ones [11]. Therefore, monitoring and quantifying the athletes’ state of fatigue in response to training/match is extremely important to take timely preventive measures. Indeed, training load monitoring allows observing whether an athlete is correctly adapting to the training program or showing signs of fatigue that could increase the risk of non-functional overreaching, injury, and illness [12]. Training load is generally classified as external and internal load. External load is defined as the work completed by the athlete, while the internal load describes the relative physiological and psychological stress imposed on the athlete [12].

In order to quantify training load, and to understand its effect on the athletes, different tools have been developed. Particularly, the use of questionnaires, diaries, and scales to monitor athletes’ psychological and physiological status was largely adopted and credited as a valid and practical method in sport [12]. The session-RPE method introduced by Foster et al. [13] and based on a modified Rating of Perceived Exertion (RPE) scale, is one of the tools widely used, also in youth soccer, to assess the internal training load (TL) by obtaining a subjective evaluation of training intensity [14]. Although considered a valid and cost-effective method, subjective measures may not be enough to identify fatigued athletes [12]. The subjectivity in the interpretation of the scale, as well as different physiological responses to training, could lead to an incorrect evaluation of the state of fatigue of the young soccer players. For this reason, different neuromuscular tests were also often employed to obtain an objective evaluation of the signals of fatigue. Isokinetic and isoinertial dynamometry, together with jump tests, became the most popular tools used in team sport environment thanks to their ease of administration and the minimal additional fatigue caused [12,15]. Therefore, a combination of subjective and objective parameters represents an optimal condition to identify athletes’ state of fatigue and the warning signs connected with an increased risk of muscle strains. To date, as reported in a recent systematic review [16], many longitudinal studies have already analyzed the association between training load, fatigue markers, and injury risk. However, there are still several limits to overcome: (1) there is a lack of specific knowledge related to youth soccer; (2) most of the studies investigated injury risk factors grouping all types of injuries together, despite ligament sprains, muscle strains, as well as other types of injuries may be characterized by a different etiology; (3) the interaction between fatigue and training load and the association with the risk of injury was not clearly investigated; (4) identifying an association does not mean being able to predict the onset of an injury; (5) many studies adopted a linear mono-dimensional approach.

To fill these limitations, it is not possible to rely on traditional linear statistical models. Differently, predictive analytics may be more suitable to achieve this scope [17]. Predictive analytics (i.e., the ability to forecast future events based on historical data) requires data mining technologies and techniques. The reason behind the adoption of data mining techniques is related to the nature of sports injuries. An injury is a complex multifactorial phenomenon determined by the interaction of several factors (modifiable and non-modifiable factors) [18]. Therefore, data mining would be helpful to detect non-trivial, non-linear, and unsuspected relations in the data [17,19]. To date, only a few studies exploited predictive analytics techniques to predict injuries. Besides, these are limited to Australian Football [20], to adult soccer players [21,22,23], or they are limited to data collected only in the preseason period [24]. Therefore, the purpose of the current study is to employ data mining algorithms to identify the hidden relationship between training load, neuromuscular fatigue, and the onset of muscle strains in young soccer players during the season. As evidenced in previous studies [25,26], maturity-related factors could influence injury predisposition in young athletes; therefore, height, body mass, and biological status of the players were also considered in this study. We hypothesized that the predictive analytic techniques could be effective in predicting the risk of muscle strains in young soccer players, as well as the combination of several factors as training load, recovery, and maturity status could modify this predisposition. 

## 2. Materials and Methods

### 2.1. Participants

Initially, twenty-three U14 soccer players were enrolled in this study. During the season, one player freely decided to withdraw from the study. Therefore, a total of twenty-two soccer players (mean ± SD: age 13.5 ± 0.3 years, body mass 51.2 ± 8.5 kg, height 164.1 ± 7.3 cm) were monitored during an entire soccer season (2018/2019). Participants were involved in a U14 sub-elite championship. They trained 3 days per week and competed once a week. All the training sessions lasted 90 min, while the matches consisted of two halves of 35 min. The data collection was obtained from the club as players’ data were routinely collected throughout the course of the season [27]. The study was conducted in accordance with the Declaration of Helsinki (2013) and approved by the local research ethics committee of the University of Rome ‘Foro Italico’ (number CAR 64/2020).

### 2.2. Injuries Data Collection

In cooperation with physical therapist and strength and condition coach, the medical team supervised injuries data collection following the Fédération Internationale de Football Association (FIFA) Consensus Statement [28]. According to this model, an injury was recorded if the player was unable to take full part in future soccer training or match [28]. For the purpose of this study, only muscle strains injuries (MSI) were included in the data mining models. Regarding their severity, MSI were classified as follows: slight (0 day); minimal (1–3 days); mild (4–7 days), moderate (8–28 days), severe (>28 days) [28].

### 2.3. Anthropometric Data, Maturity Status and Maturity Timing Estimation

Within the wide range of risk factors, several non-modifiable risk factors such as height [29] and biological maturity [30] could increase predisposition to injury in young soccer players. Therefore, anthropometric data, skeletal maturity (maturity status), and years from peak height velocity (maturity timing) were integrated into data mining models.

#### 2.3.1. Anthropometric Data

Players’ standing and sitting height were measured through a fixed stadiometer (SECA 213, measuring range 20–205 cm, SECA, Hamburg, Germany), while body mass was measured through a portable balance (SECA 762).

#### 2.3.2. Peak Height Velocity (PHV)

The Mirwald et al. [31] algorithm was employed to predict years from PHV, labelled as maturity offset (R = 0.94, R2 = 0.89, and SE = 0.59). The male specific equation was used in the current study: −9.236 + (0.0002708 × (Leg Length × Sitting Height)) + (−0.001663 × (Age × Leg Length)) + (0.007216 × (Age × Sitting Height)) + (0.02292 × (Weight/Height × 100)). Maturity offset was employed as an indicator of maturity timing (MT).

#### 2.3.3. Skeletal Maturity

Radiographs of the left-hand wrist were evaluated with the Fels method to assess skeletal maturity [32]. The radiographs were analyzed by one individual (AF) with extensive experience in these assessments. Standard errors of assessments ranged from 0.26 to 0.36 years. The difference between skeletal age (SA) and chronological age (CA) was calculated for each player and labeled as maturity status (MS). A positive value indicates that skeletal age is in advance of chronological age (early maturing players); differently a negative value indicates that skeletal age lags behind chronological age (late maturing players) [33,34].

### 2.4. Internal Load Markers and State of Recovery

The session-RPE method (S-RPE) [35] was used to quantify training and match loads of the players. S-RPE scores were obtained by multiplying the rate of perceived exertion (RPE) value, quantified through the CR-10 Borg’s scale modified by Foster et al. [35], by the duration of each training or match for every single player. Training monotony (i.e., the mean daily load divided by the standard deviation of the load over one week) and training strain (i.e., sum of weekly load multiplied by monotony) were calculated [36]. The weekly load (WL) was obtained by adding the training and matches loads over the course of a week; moreover, the cumulative loads for a period of 2, 3, and 4 weeks (WL2, WL3, WL4) were calculated.

The perceived recovery status of players was quantified using the 10-point total quality recovery scale [37]. Based on their personal psychophysical cues (e.g., mood states, muscle soreness), athletes quantified their recovery status before each training and match. The recovery status before the training (TQR) together with the previous day’s recovery status (TQR-PD) were considered in the current study.

### 2.5. Neuromuscular Fatigue

The Countermovement jump (CMJ) test is considered a practical and reliable [38] fatigue-monitoring tool used to evaluate neuromuscular status [39,40]. Therefore, CMJ was performed by the young players before (PRE-CMJ) and after (POST-CMJ) each training/match. A standardized warm-up including three minutes of light running activity, dynamic mobility exercises, and three submaximal practice jumps was executed before each testing session. At the end of the training/match, each player was re-tested within a 15-min time window. Jump height was estimated from flight time using an infrared platform (Optojump, Microgate, Bolzano, Italy). During the CMJ, the athlete was instructed to keep his hands on the hips and to jump as high as possible with no hip or knee flexion during the flight phase. The CMJs were performed to a self-selected depth. Each player executed 3 CMJs, and the highest jump was considered in the data mining models. The difference between PRE-CMJ and POST-CMJ was calculated as the percentage variation (%CMJ).

Considering that the risk of injury could change throughout the year [41], information relating to the period of the season (first, second, and third part of the season) were also included in the dataset.

## 3. Statistical Analysis

### 3.1. Injury Incidence

The MSI incidence was calculated as the number of injuries per 1000 h of play exposure.

### 3.2. Intrasession CMJ Reliability

Intrasession reliability of CMJ was assessed by comparing trial 1, trial 2, and trial 3. Relative reliability was evaluated using the intraclass correlation coefficient (ICC). According to previous studies [42], an ICC ≥ 0.70 was set as minimum acceptable reliability. In addition, absolute reliability was calculated using the coefficient of variation (CV%). CV% was calculated as the standard deviation divided by the mean score between the trials and multiplied by 100.

### 3.3. Predictive Analytics Setting

Multiple predictive models were built to predict whether a young player would get injured during the next training session based on anthropometric data, training loads, recovery status, and neuromuscular fatigue markers. Since there might be a lag between the appearance of warning signs (training load spike, state of fatigue) and the onset of injuries [43], additional models were built to evaluate the likelihood of sustaining an injury in the subsequent three training sessions, as suggested in a previous study [20], in order to explore whether the performance of models increased. Following the suggestions reported by Carey et al. [20], a lag period was added to the analysis.

### 3.4. Algorithms Selection

Different algorithms were chosen to test their ability to predict MSI in young soccer players. A set of features (Table 1), in our case the risk factors (height, body mass, MS, MT, RPE, Monotony, Strain, S-RPE, WL, WL2, WL3, WL4, TQR, TQR-PD, PRE-CMJ, POST-CMJ, %CMJ, period of the season), were inserted in the model as predictors and modeled on the binomial target variable (MSI [yes or no]). The algorithms considered were:

- Logistic Regression (LR).
- Random Forest (RF).
- Support vector machine (SVM).

Describing the underlying mathematical functions of the models is outside the scope of this paper. However, LR, largely adopted in previous studies [44,45], was selected for its ability to make simple binary classifications. RF and SVM were chosen for their ability to model complex and non-linear interactions inside high-dimensional data. The models were tested in relation to the training before injury (LR, RF, SVM) and the three training sessions before injury (LR-lag, RF-lag, SVM-lag).

### 3.5. Hyperparameters Tuning and Cross-Validation

Randomized Search method was implemented to tune hyperparameters in LR, RF, SVM, LR-lag, RF-lag, and SVM-lag. Hyperparameters were tuned using a cross-validation, and the combination of hyperparameters that returned the best performance across each fold (ROC area under the curve) was selected for further analysis. Hyperparameters tuning was performed on 20% of the dataset, and after that, these models were tested on the remaining 80% of the dataset adopting a 4-fold stratified repeated cross-validation. The entire process was repeated 1000 times to test its stability. All analysis processes were performed using Anaconda and Python libraries.

### 3.6. Data Pre-Processing

Standard pre-processing techniques were used to optimize the performance of the different models. Firstly, a data cleaning process was applied. The days in which, for any reason, players did not complete the CMJ test before and after training/match were excluded from the analysis. In addition, missing training loads data were replaced by the mean value of that player’s corresponding parameter. All the other features were normalized using Min Max Scaler (MMS). Normalization ensures that all the features fair contribution to the learning process [46]. Particularly, MMS allowed to scale down the data in a range of [−0.5, 0.5].

After the data cleaning process, the dataset showed severe class imbalance since MSI were less common compared to days when players did not get injured. Indeed, MSI represent only 2.41% of the entire dataset (days without injury = 1091, MSI = 27). To cope with class imbalance, synthetic over-sampling techniques (SMOTE) were employed. SMOTE is a combination of under-sampling (removing randomly observations from the over-represented class) and over-sampling techniques (creating new observations that have characteristics similar to already existing observations in the under-represented class) [47]. Particularly, in our study, Borderline-SMOTE, which is an extension of SMOTE, was developed. Unlike the SMOTE, where the synthetic data are randomly created, Borderline-SMOTE only over-samples the borderline minority examples [48]. Borderline-SMOTE was employed both for hyperparameters tuning and during the 4-fold repeated cross-validation, and it was applied only to the training folds.

### 3.7. Model Evaluation

**Figure 1:**
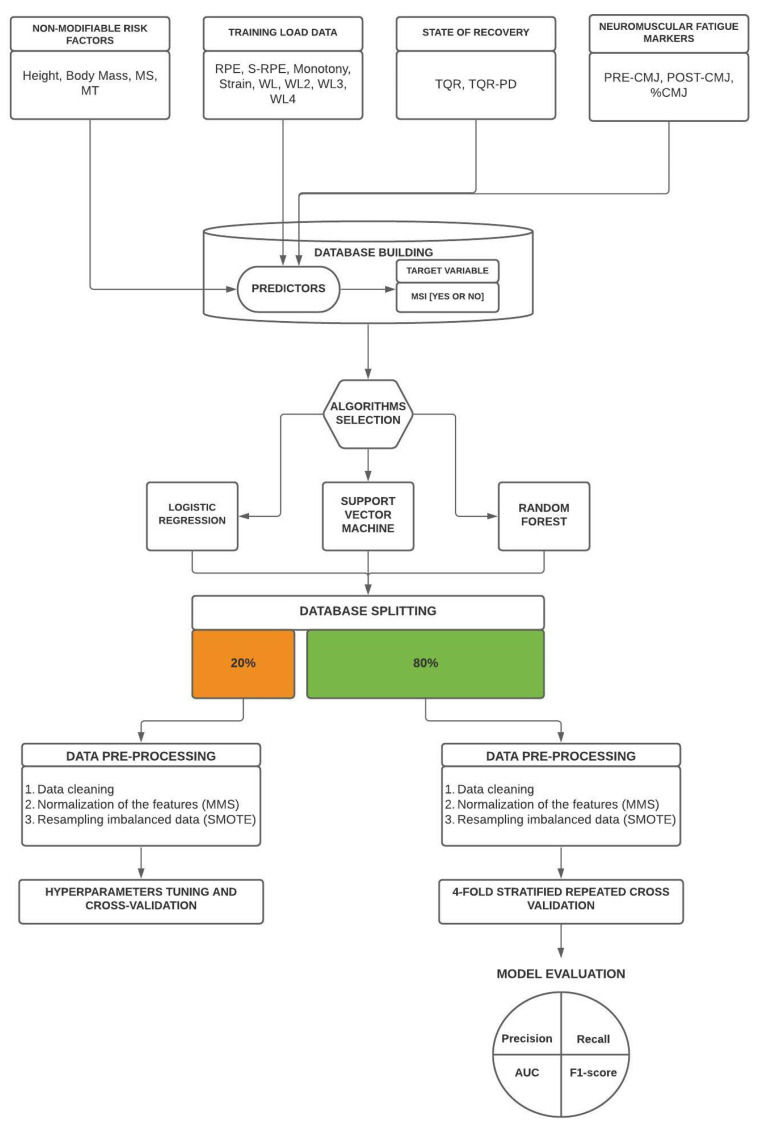
Data processing flow chart.

Accuracy can be a poor metric for an unbalanced dataset. Therefore, precision, recall and F1-score were selected to evaluate and compare the forecasting models’ goodness. Precision, calculated as follows: Precision = True Positives/(True Positives + False Positives), quantifies the number of positive observations correctly made. High precision means a lower chance of generating false positives. Instead, recall, calculated as follows: Recall = True Positives/(True Positives + False Negatives), quantifies the ability of the models to detect injuries. High recall means a lower chance of producing false negatives. F1-score is the weighted average of precision and recall. A precision and recall value equal to 1 means a 100% ability to predict the target variable. Differently, values close to zero reveal the model’s inability to work correctly. In addition, to estimate the performance of the models, a receiver operator characteristic (ROC) curve was created, and the area under the curve (AUC) was calculated. An AUC of 0.5 suggests no discrimination, between 0.51 and 0.69 poor discrimination, 0.70–0.79 acceptable discrimination, 0.8 to 0.9 is considered excellent, and more than 0.9 outstanding [49]. To get a clearer view of the procedures performed, a data processing flow chart was built and presented in Figure 1.

## 4. Results

### 4.1. MSI Incidence

A total of 40 soft-tissue injuries were registered during the entire soccer season. Among them, 27 were classified as MSI. An overall MSI incidence of 7.2 per 1000 h was found. Of the 27 injuries, 12 involved the calf (3.2 per 1000 h), 8 the thigh (2.1 per 1000 h), 6 the adductor (1.6 per 1000 h) and 1 the tibialis anterior muscle (0.26 per 1000 h). Most of MSI (44%) were classified as minimal, 9 (33%) as mild, 5 (19%) as moderate, and only 1 (4%) as severe. The number of recorded injuries (and frequency relative to the number of sessions) was reported in Table 2.

### 4.2. Intrasession CMJ Reliability

Intrasession reliability analysis revealed an excellent reliability value for CMJ (ICC = 0.87). Moreover, a small %CV was observed (4.53 ± 3.94%).

### 4.3. Predictive Analytics Models for MSI

Table 3 reports precision, recall, F1-score, and AUC to compare the performance of each model. When the models were tested considering the training before injury as the target variable, the three algorithms exhibited lower ability to predict muscle strains, as shown in Table 3. Particularly, all three algorithms (LR, RF, SVM) exhibited low precision and recall. Instead, when the models were tested on the three training sessions before an injury, the performances increased. Among the three algorithms (LR-lag, RF-lag, SVM-lag), SVM-lag showed the best performance (AUC = 0.84 ± 0.05); therefore, it was selected for further analysis.

### 4.4. Interpretation of the SVM-Lag Model

The SVM is considered one of the best supervised learning methods for classification [50] due to its ability to detect non-linear patterns. Even in our study, SVM-lag exhibited the best performance. However, the non-linear models, despite their effectiveness, are generally considered incomprehensible black-box models [51]. This could be a limitation in sport science field, where it is important to understand the causes behind a phenomenon, and to identify practical applications to share with coaches and physical trainers. To overcome this limitation and to increase the understanding of the model, a decision tree (DT) algorithm was applied to extract rules from the SVM-lag model, following the instructions reported in a previous study [51]. The original target values, used inside the original training set, were modified by the predicted values made by the SVM-lag model, and the DT algorithm was then applied to this new modified dataset [51]. The DT model allows to mimic the black-box SVM model as closely as possible, and to extract human-comprehensible rules [51].

**Figure 2:**
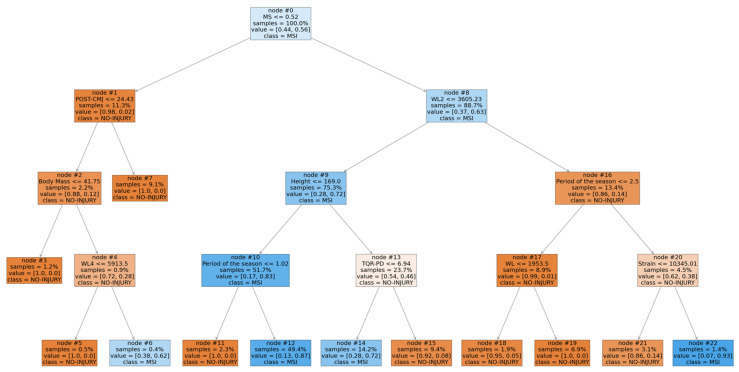
Decision tree model.

The resulting DT model was presented in Figure 2. A DT is a directed acyclic graph consisting of a combination of internal nodes and leaf nodes. Each internal node presents a specific test that must be carried out on a single variable. In relation to the test result, the branches indicate the possible outcomes. Therefore, it is possible to classify the different observations starting from the root node and following the path towards the leaf nodes. In our specific case, the orange leaf nodes indicated no risk of MSI. Differently, the blue leaf nodes marked the increased risk of MSI. Within the current study, we analyzed the conditions that, according to the rules extracted from the SVM-lag model, could increase the risk of MSI.

The root node of the model split according to MS. An advanced maturity status (MS > 0.52 years) determined a higher risk of MSI (0.44% vs. 0.56% observations, node 0).

For late maturing players, the following risk factors were identified:

- POST-CMJ (≤24.43 cm, node 1) combined with a high body mass (>41.75 kg, node 2) and a high WL4 (>5913.5 AU, node 4).

For early maturing players, the following risk factors were identified:

- A low WL2 (≤3605.23 AU, node 8) combined with a high height (>169 cm, node 9) and a low recovery status (TQR-PD ≤6.94 AU, node 13).
- A low WL2 (≤3605.23 AU, node 8) combined with a low height (≤169 cm, node 9) during the second and third part of the season (node 10).
- A high WL2 (>3605.23 AU, node 8) during the third part of the season (node 16) and combined with a high strain (>10,345.01 AU, node 20).

## 5. Discussion

The main purpose of this study was to exploit predictive analytics techniques to predict MSI in young soccer players. To date, the interaction between fatigue and training load and the association with the risk of injury has not been clearly investigated. Therefore, data mining algorithms were employed in the current study to fill this gap. In accordance with our hypotheses, predictive analytic techniques proved to be effective in identifying MSI and understanding the underlying risk factors.

Among the different supervised learning techniques selected, the SVM produced the best performance (AUC = 0.84 ± 0.05); instead, LR and RF showed less predictive ability. As a linear model, LR, widely adopted in previous studies [44,52] may not be suited to recognize non-linear relationships [53]. Differently, RF being a more complex model, tends to suffer from overfitting and thus be unable to predict injuries in the test set [20].

A similar approach was developed by two different studies of López-Valenciano et al. [54] and Rossi et al. [22], who nevertheless investigated the risk of injury in adult male professional soccer players. In the first study, the authors built forecast models based only on personal, psychological, and neuromuscular measures collected during the pre-season [54]. In the second study, the authors monitored 26 professional male players for 23 weeks and used daily workload data to build a general non-contact injury model [22]. As mentioned above, the various types of injuries could be characterized by a different etiology; therefore, the current study focused only on MSI. To the best of our knowledge, this is the first study that exploited data mining algorithms to predict MSI risk in young soccer players during a soccer season, combining training load and neuromuscular fatigue markers. The SVM of this study exhibited the best performance, but only when the three training sessions before MSI were included in the model (SVM-lag). Therefore, considering only the information collected the day before an injury may not be enough to identify injury risk factors. In line with this assumption, Hulin et al. [43] found a greater risk of injury in the week following a spike in the training load. Therefore, signs of fatigue might appear a few days before the onset of an injury. For this reason, daily monitoring allows to promptly identify warning signals and to promote preventive strategies.

Although the SVM-lag model exhibited good performance in identifying the risk of MSI, it is considered an incomprehensible black-box model [51]. To overcome this limitation, a rule extraction technique adopting a DT algorithm was developed. The model was presented in Figure 2. It is possible to observe that the various factors interact with each other modifying the susceptibility to injury, confirming that MSI are a complex multifactorial phenomenon. The root node of the DT model split based on the MS. Particularly, an advanced maturity status (MS > 0.52 years) produced a higher risk of injury, as evidenced by the different numbers of observations recorded in the two branches (0.44% vs. 0.56%). This result aligns with a previous study [55], where a higher incidence of MSI was reported in more mature players. The more mature players are able to maximize the use of anaerobic system [56] and consequently express more power, speed and strength. Moreover, they are characterized by a greater body mass that could increase the risk of lower limb muscle strains [57]. Indeed, more mature players owing to their body size, produce a greater match running performance compared to their less mature teammates [57]. All these factors could increase susceptibility to MSI in more mature players being involved in a more demanding context. At this stage, the interaction between training load and neuromuscular fatigue markers modified susceptibility to MSI. For late maturing players (left branch) the root node produced a decision node that split according to the POST-CMJ (node 1). A low POST-CMJ (≤24.43 cm, node 1) combined with a high body mass (>41.75 kg, node 2) and a high WL4 (>5913.5 AU, node 4) increased the risk of injuries. Jump tests are often adopted in team sport environment to quantify neuromuscular fatigue [12]. Therefore, a low CMJ score after the training could indicate a fatigue condition. The state of fatigue, and consequently the greater risk of injury, could also be determined by an excessive training load as evidenced by node 4 (WL4 > 5913.5 AU). The threshold value identified by the DT model is higher compared to the average value recorded during the season (5486 AU; Table 1). In line with previous studies [58,59], this underlines that high loads maintained for longer periods may increase the state of fatigue and risk of injury. Furthermore, the risk of injury appears to be higher for the players with a body mass greater than 41.75 kg (node 2), confirming the above mentioned.

The role of training load in preventing or increasing the risk of injury is further highlighted by analyzing the right branch of the DT model. Indeed, a WL2 higher than 3605.23 AU (node 8), cumulated during the latter part of the season (node 16) and associated with a very high strain (>10,345.01 AU, node 20) increased the risk of MSI. Even in this case, the training load parameters are higher than the average values recorded during the season (Table 1). In addition, a further important aspect was highlighted: the risk of injury increases during the latter part of the season. In accordance with Malone et al. [58], coaches and medical staff should be aware that players tolerate workloads differently in relation to the period of the season, as also highlighted by node 10. Among the other risk factors identified, a low WL2 (≤3605.23 AU, node 8) combined with a lofty height (>169 cm, node 9) and low TQR-PD values (≤6.94 AU, node 13) were recognized as a dangerous condition for MSI (0.28% vs. 0.72%). The impact of stature on injury risk has been investigated in previous studies [29,60,61]. The authors suggest that the higher biomechanical load [61] or the poor motor coordination [29] could explain the higher injury risk in taller players. This risk could further increase in conditions of low recovery status, as evidenced by node 13 (TQR-PD ≤6.94 AU). A low recovery status was also recognized as an injury risk factor in previous studies [26,62]. As highlighted by Kenttä & Hassmén [63], an imbalance between recovery and training load may challenge athletes’ stress tolerance increasing predisposition to injury.

Through the analysis of the DT, it was possible to observe how numerous factors (non-modifiable factors, neuromuscular fatigue, training load), interacting with each other in a non-linear manner, modify predisposition to MSI. The complexity in managing the training load lies in the fact that both a lower workload (node 8) or a higher workload (node 4, node 20) can increase susceptibility to injury, and this susceptibility could also change in relation to the period of the season. In summary, the rules extracted through the DT algorithm, in accordance with previous studies, show that:

- Risk of injury could change according to the maturity status of the players.
- Monitoring CMJ before and after training could be a useful tool to identify a greater state of fatigue and, therefore, a higher predisposition to injury.
- The susceptibility to injury could be modified by anthropometric factors (body mass and height).
- A high workload and a low players’ recovery status could increase predisposition to muscle injuries.

**Figure 3:**
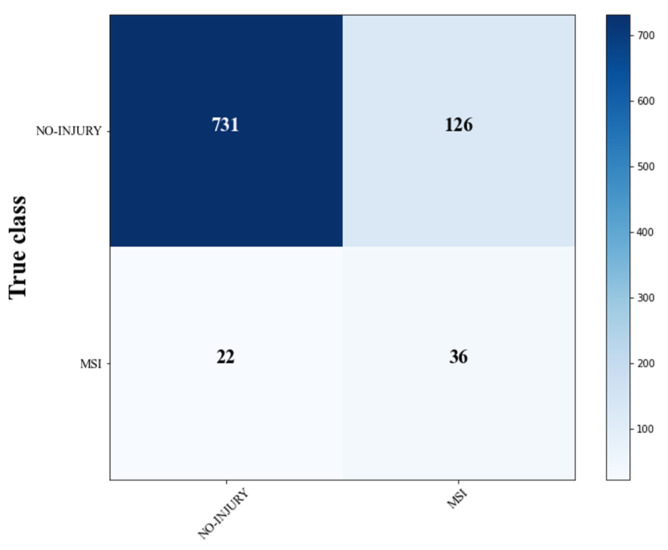
SVM-lag confusion matrix.

Although the SVM-lag model showed a good performance in identifying players at risk of MSI, and the DT allowed us to understand the complex interactions between training load and neuromuscular fatigue, the study presents some limitations and needs several considerations. First of all, as reported in the confusion matrix created after the cross-validation (Figure 3), the model was able to predict more than half of the injury risk conditions correctly, however producing a high number of false positives. That explains the low precision identified (Table 3). Although false positives may produce a less clinical impact compared to false negatives [64], a high number of false alarms may lead a coach to “stop” an athlete several times, increasing the time-loss from sports participation. Moreover, the study is limited to U14 soccer players. Therefore, to increase the generalization capacity of the model developed, future studies should include a larger sample size involving players of different chronological ages. That would also allow increasing the number of injuries collected and the predictive ability of the model. Furthermore, combining internal load data with external load data would improve the ability to correctly quantify the weekly training load and accurately identify fatigue signs.

However, adopting predictive analytics techniques sounds promising as it would allow to analyze the complex interactions between numerous features and move towards a multi-dimensional approach.

## 6. Practical Applications

**Figure 4:**
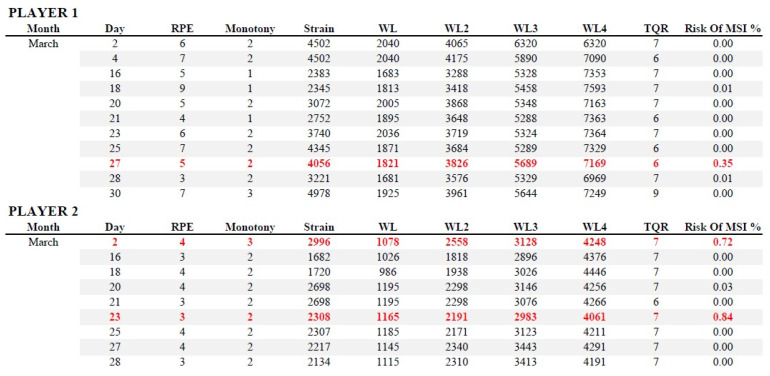
Example of the daily workload of two players and the relative estimated injury probability (Risk Of MSI%). In red the days with higher risk.

To date, predicting MSI with high accuracy represents a complex task due to the multifactorial nature of injuries. This task is even more complex if we try to predict muscle injuries only by relying on data recorded during the previous training. Indeed, as reported in our study, the predictive ability of data mining models has increased considering the three trainings prior to an injury. For this reason, coaches and physical trainers should encourage constant daily monitoring. Considering the complexity of predicting an injury with high accuracy based only on the previous day’s data, it could be useful to evaluate day by day the injury probabilities predicted by the model and then implement prevention strategies. In Figure 4, an example of the daily workload of two players and the relative estimated injury probability was presented. Coaches and physical trainers should evaluate the extent of the predicted injury risk (from 0% to 100%) and implement adequate prevention strategies: training load reduction, recovery strategies, or individualized training sessions.

## 7. Conclusions

The main purpose of this study was to compare the ability of different predictive analytic techniques to predict the risk of MSI in young soccer players. The data mining algorithms allowed to evaluate how the interactions of non-modifiable factors (anthropometric data, MS, MT), training load parameters, and neuromuscular fatigue markers modified predisposition to injury. Among the different algorithms adopted, the SVM-lag model produced the best performance (AUC = 0.84 ± 0.05), showing the highest precision and recall. The SVM-lag model allowed to identify non-linear interactions between training load, neuromuscular fatigue, and the onset of muscle strains in young soccer players. Instead, the adoption of the DT allowed to understand the interactions identified by the SVM-lag model. Particularly, the risk of MSI in young soccer players could change in relation to maturity status and anthropometric factors. Moreover, a higher neuromuscular fatigue and workload, together with a lower recovery status could increase their susceptibility to injury.

This information may be used by coached and physical trainers to understand the factors that could lead to a MSI, and consequently to manage the weekly training load, to detect signs of fatigue, and to develop adequate prevention strategies. Future studies should verify whether the use of predictive analytics techniques allows to reduce the incidence of injuries during the season.

## Figures and Tables

**Table 1:** Summary of the features inserted the data mining models together with the average values calculated during the entire season.

Variables	Definition	Collection/Calculation	Average Values
Maturity timing (MT)	Years from peak height velocity (PHV)	Mirwald et al. [31] algorithm	−0.2 ± 0.66 years
Maturity status (MS)	Level of maturation at the chronological age (CA) of observation	Skeletal age (SA) − CA	1.09 ± 1.04 years
RPE	Rate of perceived exertion	CR-10 Borg’s scale modified by Foster et al. [35]	4.6 ± 1.89 AU
S-RPE	Subjective internal training load (TL)	RPE× Training duration	426.8 ± 283.1 AU
Monotony	Statistical analysis of trainings’ variation over time	$\frac{\mathrm{Mean}\;\mathrm{daily}\;\mathrm{load}}{\begin{array}{c} \mathrm{Standard}\;\mathrm{deviation}\;\\ \mathrm{of}\;\mathrm{weekly}\;\mathrm{TL} \end{array}}$	2.96 ± 2.96 AU
Strain	Overall stress of the training week	Monotony× sum of weekly TL	4613 ± 4008 AU
WL	Cumulative loads for a period of one week	Sum of the loads of all training/match sessions over a period of one week	1679 ± 1043 AU
WL2	Cumulative loads for a period of two weeks	Sum of the loads of all training/match sessions over a period of two weeks	3126 ± 1717 AU
WL3	Cumulative loads for a period of three weeks	Sum of the loads of all training/match sessions over a period of three weeks	4325 ± 1843 AU
WL4	Cumulative loads for a period of four weeks	Sum of the loads of all training/match sessions over a period of four weeks	5486 ± 2028 AU
TQR	Recovery status before the training session	TQR scale	7 ± 1.49 AU
TQR-PD	Previous day’s recovery status	TQR scale	7 ± 1.47 AU
PRE-CMJ	Jump height assessed before the training session	Infrared platform (Optojump system)	31.25 ± 5.30 cm
POST-CMJ	Jump height assessed after the training session	Infrared platform (Optojump system)	30.60 ± 5.09 cm
%CMJ	Percentage variation between PRE-CMJ and POST-CMJ	$\frac{\left(\mathrm{PRE}-\mathrm{CMJ}\right)-\left(\mathrm{POST}-\mathrm{CMJ}\right)}{\mathrm{POST}-\mathrm{CMJ}}\times 100$	−1.60 ± 9.28%

Data are expressed ad mean ± SD.

**Table 2:** Number of MSI and rates relative to the number of sessions.

Injury Outcome	Number of Injuries (Frequency)
MSI	27 (0.024)
MSI-lag	64 (0.057)
Total sessions (trainings and matches)	1118

**Table 3:** Performance of the data mining models analyzing the training before injury (LR, RF, SVM) and three training sessions before injury (LR-lag, RF-lag, SVM-lag). Precision, Recall, F1-score and the overall AUC were reported. mean and the standard deviation of the evaluation metrics over 1000 cross validation tasks.

Models	Condition	Precision	Recall	F1-Score	AUC
LR	NI	0.97 ± 0.01	0.97 ± 0.02	0.97 ± 0.01	0.63 ± 0.09
	MSI	0.04 ± 0.1	0.05 ± 0.09	0.04 ± 0.08	
RF	NI	0.97 ± 0.01	0.98 ± 0.01	0.98 ± 0.01	0.58 ± 0.14
	MSI	0.03 ± 0.12	0.03 ± 0.08	0.03 ± 0.08	
SVM	NI	0.98 ± 0.01	0.86 ± 0.03	0.91 ± 0.02	0.55 ± 0.16
	MSI	0.04 ± 0.03	0.2 ± 0.16	0.06 ± 0.05	
LR-lag	NI	0.95 ± 0.01	0.75 ± 0.05	0.84 ± 0.03	0.66 ± 0.07
	MSI	0.1 ± 0.04	0.39 ± 0.17	0.15 ± 0.06	
RF-lag	NI	0.95 ± 0.01	0.85 ± 0.07	0.89 ± 0.04	0.71 ± 0.07
	MSI	0.12 ± 0.07	0.29 ± 0.17	0.16 ± 0.08	
SVM-lag	NI	0.97 ± 0.01	0.86 ± 0.03	0.91 ± 0.02	0.84 ± 0.05
	MSI	0.21 ± 0.05	0.55 ± 0.14	0.3 ± 0.07	

NI = no-injury; MSI = muscle strain injuries; LR = Logistic Regression; RF = Random Forest; SVM = Support Vector Machine.

## Data Availability

Data are available upon reasonable request.
